# Remarks upon Puerperal Fever Complicated with Malaria

**Published:** 1849-01

**Authors:** Edward R. Parks

**Affiliations:** Leesburg, Indiana


### ARTICLE II.

*Remarks upon Puerperal Fever Complicated with Malaria.* Read before the Kosciusko County Medical Society. By EDWARD R. PARKS, M. D., of Leesburg, Indiana.

The earliest history of medicine gives accounts of puerpal fever, and modern authors have described it with great accuracy and precision. At one time, the disease was supposed to consist essentially in inflammation of the uterus, its appendages, its peritoneal investing membrane, or in inflammation of the peritoneum generally; or in all these simultaneously. But it is now generally conceded that puerperal fever depends also upon other and very different pathological conditions than inflammation.

It may depend upon simple engorgement and congestion of the uterus independent of inflammation.

Uterine phlebitis is frequently the cause of puerperal fever.

It may also depend upon disease or derangement of the function of the lymphatics of the uterus.

If these premises are true, puerperal fever does not depend on any one specific cause, and, consequently, is merely a common name for several dissimilar pathological conditions occurring during the puerperal state. But there is one form of peurperal fever which is very common, (in this country, at least) that has not been described by any author. We allude to this disease complicated with malaria, or, malarial disease complicated with the puerperal state.

We have, for the last few years, had our attention directed to the investigation of this disease, and having had a pretty extensive opportunity of seeing diseases of this description, and we have no hesitation in saying that a large majority of the deaths among the child-bearing females of this country may be justly attributed to this disease.

There are two distinct varieties of this malady, depending for the most part upon the season of the year and the prevailing type of disease. During the summer and early part of the fall it will assume a more or less inflammatory character. But during the winter and early part of the spring, the disease will certainly put on a decidedly typhoid character.

There is, probably, very little observable difference in the symptoms of this complicated form, and the ordinary disease of the books.

In the sthenic variety which is met with during the summer and early part of the fall, the symptoms may be precisely such as are described by most of our modern authors, and yet the case may be complicated with malaria.

How, then, can we be enabled to form a correct diagnosis?

This is the only question of much moment in the case. And, although it is one attended with some difficulty, yet, by having our attention properly directed to the probability of such complication, we may be always enabled to form a correct diagnosis.

In the first place, if our patient has suffered with any malarial disease during gestation, especially if it should be of recent date, we are justified in the conclusion that the disease is complicated with malaria; or should we only find that the patient had, during the last year or so, suffered with malarial disease, and that she is now laboring under its sequelæ, we may strongly suspect the complication.

Our diagnosis will also be materially aided by the extent of malarial disease prevailing at the time. Should any of the various forms of this unseen enemy be extensively prevailing in the neighborhood, you have strong grounds for suspecting its influence in the case. But should all these conditions, or several of them, exist, you have the strongest possible grounds, and may predict with absolute certainty, that this complication does exist. With reference to the asthenic or typhoid variety, we can only, in this place, hint as we pass. Much of what we have just said, in reference to the sthenic variety, will apply with equal force, also, in this form of the malady; especially so much as related to the pre-existence of malaria in the system, and to the extent of its prevalence in the country at the time. This form of the disease, it will be recollected, is met with during the cold and variable seasons, as winter and spring. Now, if you have a patient during this season of the year, attacked with puerperal fever, and asthenic pneumonia is prevailing extensively, the disease will certainly assume the typhoid type, more especially if the patient should have suffered with a recent intermittent. But whether she should have suffered such an attack or not, if asthenic pneumonia is prevailing, we may conclude, with almost positive certainty, that the fever is complicated with malaria, and will absolutely put on a decidedly typhoid form. The disease will also frequently be complicated with pneumonia.

*Symptoms.*—The disease often commences some time previous to accouchment. In this instance, which is the most usual manner of its approach, its invasion is very insidious. The patient will be seized with a violent catarrh; this is attended with a troublesome cough, some pain about the chest, with dyspnœa and a state of pyrexiæ, attended with manifest exacerbations and remissions.

These symptoms are regarded by the patient and her friends as "only a bad cold," and, consequently, attended with little danger. If medical advice is not sought, and the disease continues, the patient becomes debilitated and anæmic. The time of her accouchment arrives. She may have the most natural and easy labor, or otherwise, but this slight pyrexiæ continues: the cough frequently becomes aggravated; and the dyspnœa greater; the pulse, which had been feeble and quick, becomes gradually quicker and quicker, until it rises from a hundred and twenty to a hundred and sixty. The whole surface is remarkably pale; the cheeks and prolabia are decidedly anæmic and exsanguinous. Little or no pain is complained of by the patient. There is no tumefaction, and but very little, if any, tenderness over the abdomen; pain, or a sense of throbbing, in the head is frequently complained of by the patient. This is attended with perverted action of the whole arterial system; the carotids can frequently be observed throbbing from a considerable distance. Delirium is a very frequent symptom. Jactitation and subsultus tendinum are also very common.

For a more particular history of the symptoms of puerperal fever, we must refer to systematic authors.

*Pathology.*—Our own opinion in relation to the proximate cause of this disease we will briefly state in a few words. From what has already been said it will be seen that we regard malaria both as a predisposing and exciting cause of this malady, consequently, all that is necssary to the full development of the disease, is the puerperal state complicated with malaria. Hence it is that few puerperal women, who are under the malarial influence, escape this disease. As to the particular or special primary impression made upon the system, we believe (with many others,) that the whole nervous system is the true seat of this disease. But concerning the peculiar nature of the primary impression on the nerves, and the many hypotheses that have been entertained, we have neither space nor inclination to dilate.

*Treatment.*—Bloodletting stands at the head of the treatment of puerperal fever, as described in the books. But we only allude to it in this place to proscribe it as a remedy in this form of puerperal fever. We have never, in a solitary instance, resorted to the use of the lancet in this disease. It might, however, in some rare instances, be used with advantage. But it should, certainly, be resorted to with great circumspection. As a general rule we regard it as decidedly prejudicial.

Cathartics are sometimes useful in the more sthenic forms of the disease. When there is torpor of the bowels, as sometimes happens, with the tongue loaded with a dense yellowish or brownish coat, a mercurial cathartic will afford considerable relief. But by far the most important remedies in the treatment of this affection, are the disulphate of quinine, morphine, and vesicants.

In this, as in all other forms of malarious disease, the disulphate of quinine is the great antidote. It should be combined with morphine in proportions of from five to ten grains of the quinine, to from one third to a whole grain of the morphine. This should be repeated every two or three hours until an obvious impression is made on the disease. In some instances, morphine will be found to produce some unpleasant effects. In such cases the extract of hyosciamus or cicuta, will have an admirable effect. They should be given in doses of from two to six grains every two or three hours until the patient is brought perfectly under their influence.

In the more asthenic forms of the disease, the patient's strength must be supported from the commencement, or she will certainly sink under the influence of the malady.

Quinine, wine, and even brandy, are to be freely and fearlessly administered for this purpose.

Blisters cannot be used with propriety in the decidedly asthenic or typhoid form of this disease. But in the more sthenic forms, where there is some tumescence of the abdomen, attended with pain and tenderness, they are of the greatest value and should not be omitted. They should be made to cover the whole abdomen.

*Prophylactics.*—Although we have not much faith in prophylactics in general, yet, in this disease they are of infinite importance. Much may be done in this way by the accoucheur. He frequently sees his patient, or is consulted, previously to accouchment. It is certainly his duty, when he finds any of the predisposing causes of this disease existing, to warn the patient or her friends of her danger; for, if he does not, she is almost certainly doomed. Of course, when there is any evidence of the existence of malaria in the system, it can be safely and successfully treated with the great antidote. The cough, dyspnœa, etc. may be effectually relieved by an occasional dose of morphine and ipecac.

We should have been glad to have entered somwhat more in detail upon the treatment, but our limits would not permit It is, certainly, a disease that we have seen much of, and we have thus briefly glanced at the kind of treatment that we have often witnessed, crowned with the most salutary and happy effects.

